# Differences in CD24 Expression Between Prostate Adenocarcinoma and Benign Prostatic Hyperplasia: A Cross-sectional Study

**DOI:** 10.30699/ijp.2024.2021959.3251

**Published:** 2024-07-24

**Authors:** Mahdi Sajedifar, Atieh Jafarabadi Ashtiani, Mohammadreza Jalali Nadoushan

**Affiliations:** 1 Student Research Committee, Faculty of Medicine, Shahed University, Tehran, Iran; 2 Department of Pathology, Faculty of Medicine, Shahed University, Tehran, Iran

**Keywords:** BPH, CD24, Gleason grade, Prostate adenocarcinoma

## Abstract

**Background & Objective:**

*CD24* is a small, highly glycosylated membrane protein whose expression is associated with tumorigenesis and the progression of several types of cancer. Prostate adenocarcinoma is one of the most common cancers in men, and microscopic Gleason grading is an important factor affecting prognosis. This study aims to investigate the relationship between immunohistochemical expression of *CD24* and its relationship with benign prostatic hyperplasia (BPH) and Gleason grade in prostate adenocarcinoma.

**Methods:**

This cross-sectional study was conducted on 163 patients, with an average age of 70.63±9.05 years, including 78 (47.9%) patients with prostate adenocarcinoma and 85 (52.1%) patients with benign prostatic hyperplasia., referred to Mostafa Khomeini Hospital in Tehran between 2018 and 2021, who underwent open prostatectomy or Trans Urethral Resection of Prostate (TURP). Immunohistochemical staining was used to evaluate *CD24* expression, and Gleason grade was determined in the case of prostate adenocarcinoma. Data were analyzed with SPSS 22 and a P-value<0.05 was considered statistically significant.

**Results:**

The percentage and intensity of *CD24* staining in prostate adenocarcinoma patients was significantly higher than in BPH patients (*P*<0.05). Gleason score strongly correlated with the percentage and intensity of *CD24* staining (*P*<0.05). The immunoreactive score, obtained by multiplying the *CD24* expression percentage with staining intensity, was also significantly related to the Gleason score (*P*<0.05).

**Conclusion:**

*CD24* expression can be considered as a factor in differentiating cases of prostate adenocarcinoma from benign prostatic hyperplasia. Also, a high level of this marker can indicate the progress of prostate cancer.

## Introduction

The concept of biomarkers, defined as laboratory measurements intricately linked to pathologic processes and possessing diagnostic and prognostic capabilities (1), has been pivotal in the realm of medical diagnosis and prognosis (2, 3). Prostate cancer (PCa), a complex entity with a highly variable clinical course, has long relied on biomarkers, notably prostate-specific antigen (*P*SA), for diagnostic purposes over the past two decades (4, 5). However, despite the longstanding use of PSA, its inadequacies in differentiating the aggressiveness of PCa have become evident (6). PCa, with its unpredictable clinical trajectory, poses challenges in accurate risk stratification (7). While prostate biopsy remains the gold standard for diagnosis, its limitations and invasive nature elevate the risk of adverse events (8). Moreover, reliance on PSA, prostate biopsy Gleason score, or pTNM cancer stage for risk stratification may lead to potential understanding (7). Accurate staging and risk stratification become paramount, particularly in the context of active surveillance (9). Consequently, there emerges a pressing need for refined PCa biomarkers that not only exhibit enhanced accuracy but also possess superior risk stratification properties. 

One promising avenue of exploration in this quest for improved biomarkers involves the investigation of *CD24*, initially identified as a marker for B cells (10). Subsequent research has unveiled the expression of *CD24* in various hematological malignancies and solid tumors, including lung cancer, neuroblastoma, rhabdomyosarcoma cells, and renal cell carcinoma (11-14). Notably, *CD24*'s positivity and staining pattern have emerged as significant molecular markers for diagnosing malignancy and predicting lymph node metastasis (15). In the specific context of prostate cancer, limited studies have delved into the relationship between CD24 expression levels and prognosis (12, 13, 16). Furthermore, a notable gap in the literature pertains to the exploration of *CD24* gene expression variations in tumors exhibiting equal Gleason grade but disparate primary and secondary pathological views. This lacuna underscores the need for a comprehensive investigation into the expression of the *CD24* marker and its correlation with benign prostatic hyperplasia and Gleason grade in prostate adenocarcinoma.

In light of these considerations, this study aims to bridge existing gaps in knowledge by thoroughly examining the expression of the *CD24* marker and its nuanced relationship with benign prostatic hyperplasia and Gleason grade in prostate adenocarcinoma. The overarching goal is to contribute to the identification of improved PCa biomarkers that not only enhance accuracy but also refine the risk stratification process. Such advancements are imperative to address the critical need for markers capable of distinguishing indolent from aggressive cancers, thereby mitigating the risk of overtreating prostate cancer—a goal of paramount significance in the ongoing pursuit of precision medicine in oncology.

## Material and Methods

This cross-sectional study involved 163 patients, comprising 78 individuals with prostate adenocarcinoma and 85 patients with benign prostatic hyperplasia (BPH). The subjects were drawn from those referred to Shahid Mostafa Khomeini Hospital in Tehran between 2018 and 2021, all of whom had undergone either open prostatectomy or Trans Urethral Resection of the Prostate (TURP). The inclusion criteria encompassed all samples, with no exclusions made. Cases meeting the exclusion criteria involved instances where samples exhibited extensive necrosis and hemorrhage, rendering them unsuitable for immunohistochemical (IHC) analysis. It is noteworthy that no such cases were encountered. Upon obtaining approval from the local ethics committee for the study, the sample was collected. Information about macroscopic characteristics, including tumor size (maximum diameter) and patients' age, was derived from their medical records. The hematoxylin and eosin-stained (H&E) tissue specimens were examined by a pathologist to determine the tumor type and Gleason grade. In addition, 3-micrometer sections were stained by immunohistochemical method during the following steps to evaluate *CD24* expression. Unexaminable slides and inconvenient tissue samples were excluded from the study. The slides were placed at 60°C for 1 h, and after melting the paraffin, the slides were exposed to xylol for 10 min and alcohol (concentrations of 100%, 96%, and 70%) for 5 min. For 10 minutes, 3% hydrogen peroxide was applied to the slides and then washed off. Citrate buffer (pH=6) was poured on the slides and placed in an autoclave at 120˚C and 1.2 atm for 10 min. The slides were washed with TBS buffer (pH=7.2-7.6), then one drop of peroxidase blocking solution was poured on the slides, followed by twice washing with TBS after 5 min. The protein-blocking solution was subjected to the same steps. These actions were designed to eliminate the excess antibodies.

The next step was to cover the slides with CD24 antibody and wash them twice with TBS buffer after 1 hour. Hematoxylin dye was used to soak the slides for 1 min, and then the tissues were dehydrated with alcohol at a concentration of 60, 97, and 100%. Then, the section clearing was conducted using xylol. Sections were evaluated by a pathologist for *CD24* expression in tumor cells. The categorization of *CD24* expression percentage was delineated into five distinct categories: zero, indicating a lack of staining; 1) denoting less than 10% of positive cells; 2) representing 11 to 50% of positive cells; 3) encompassing 51 to 80% of positive cells; and 4, indicating 80% to 100% positive cells (12). The staining intensity was determined from 0 (-) to 3 (+++). Multiplying the percentage of *CD24* expression by the intensity of CD24 expression was calculated as the Immunoreactive score for each sample, which indicated values above 100 as high expression and below 100 as low expression (16). The data underwent statistical analysis with a significance level set at 0.05, utilizing SPSS version 22 (SPSS Inc., Chicago, IL., USA). The statistical analysis encompassed the application of independent t-tests, one-way analysis of variance (ANOVA), and Chi-square tests.

## Results

**Fig. 1:**
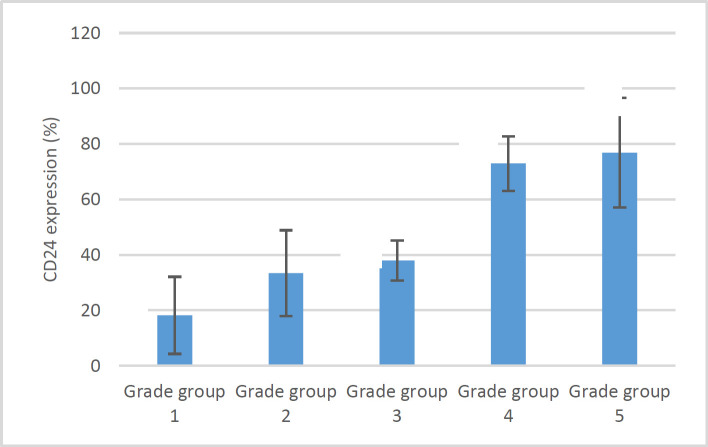
CD24 expression in relation to Gleason grade Different symbols indicate statistically significant differences.

In the present investigation, a cohort of 163 male individuals with a mean age of 70.63±9.05 years underwent examination. This group comprised 78 individuals (47.9%) diagnosed with prostate adenocarcinoma and 85 individuals (52.1%) diagnosed with benign prostatic hyperplasia (BPH). Analysis using the independent t-test revealed no statistically significant difference in age between the two groups (*P*=0.955). The mean percentage of CD24 expression (*P*ercentage of stained cells) in patients with prostate adenocarcinoma (36.69±27.21%) was significantly higher than that of BPH patients (11.61±10.68%) (*P*<0.001). Also, the *CD24* staining intensity in the prostate adenocarcinoma group (1.87±1.02) was significantly higher than that of BPH patients (0.90±0.78) (*P*<0.001). CD24 expression in more than 50% of cells was observed in 28.2% of patients with prostate adenocarcinoma, whereas none of the patients with BPH showed an expression percentage higher than 50%. The Chi-square test results indicate a statistically significant difference (*P*<0.001). Also, an immunoreactive score of more than 100 was reported in 33.3% of prostate adenocarcinoma patients and 0% of BPH patients, and a significant difference was observed between the two groups (*P*<0.001) (Table 1). Gleason grade is specific for patients with prostate adenocarcinoma, and more than half of these patients (52.6%) had Gleason grade group 1, which indicates a Gleason score of 6 or less. After that, grade group 4 had the highest prevalence in prostate adenocarcinoma patients (19.2%). No substantial correlation was observed between age and the Gleason grade groups (*P*=0.204). Findings from the one-way ANOVA test indicated the *CD24* expression and staining intensity are significantly different between various Gleason grade groups (*P*<0.001). Duncan's post hoc test showed that *CD24* expression was significantly lower in samples with grade group 1 and significantly higher in samples with grade groups 4 and 5 than others (Figure 1). The findings indicate that grades groups 1 and 2 have a significantly lower *CD24* staining intensity than higher grades. According to the Chi-square statistical test, increasing the Gleason grade leads to a significant increase in *CD24* expression and Immunoreactive scores (*P*<0.001) (Table 2). Gleason score had a strong and positive correlation with the percentage and intensity of *CD24* staining (*P*<0.05).

**Table 1:** Characteristics and results of pathological evaluation of specimens

		Total	BPH	Adenocarcinoma	P-value
Age (year)		70.63±9.05	70.67±9.23	70.58±8.91	0.955
Staining intensity		1.37±1.02	0.90±0.78	1.87±1.02	<0.001
CD24 expression (%)		23.61±23.85	11.61±10.68	36.69±27.21	<0.001
Immunoreactive Score	High (>100)	26 (16.0%)	0 (0.0%)	26 (33.3%)	<0.001
	Low (≤100)	137 (84.0%)	85 (100%)	52 (66.7%)	
CD24 positive cells	0%	41 (25.2%)	30 (35.3%)	11 (14.1%)	<0.001
	≤10	13 (8.0%)	11 (12.9%)	2 (2.6%)	
	11%-50%	87 (53.4%)	44 (51.8%)	43 (55.1%)	
	51%-80%	15 (9.2%)	0 (0.0%)	15 (19.2%)	
	>80	7 (4.3%)	0 (0.0%)	7 (9.0%)	

**Table 2:** Relationship between Gleason grade and basic/pathological characteristics

		Gleason grade groups					*P*
		Grade group 1 (n=41)	Grade group 2 (n=10)	Grade group 3 (n=6)	Grade group 4 (n=15)	Grade group 5 (n=6)	
Age (year)		71.97±9.30	65.20±7.55	67.83±11.97	72.20±5.07	68.83±10.88	0.204
Staining intensity		1.34±0.91	1.70±0.94	2.50±0.54	2.86±0.35	2.66±0.81	<0.001
Immunoreactive Score	High (>100)	2 (4.9%)	2 (4.9%)	2 (33.3%)	15 (100%)	5 (83.3%)	<0.001
	Low (≤100)	39 (95.1%)	8 (80.0%)	4 (66.7%)	0 (0.0%)	1 (16.7%)	
CD24 positive cells	0%	10 (24.4%)	1 (10.0%)	10 (24.4%)	0 (0.0%)	0 (0.0%)	<0.001
	≤10	2 (4.9%)	0 (0.0%)	2 (4.9%)	0 (0.0%)	0 (0.0%)	
	11%-50%	29 (70.7%)	8 (80.0%)	29 (70.7%)	0 (0.0%)	1 (16.7%)	
	51%-80%	0 (0.0%)	1 (10.0%)	0 (0.0%)	11 (73.3%)	2 (33.3%)	
	>80	0 (0.0%)	0 (0.0%)	0 (0.0%)	4 (26.7%)	3 (50.0%)	

٭-indicate statistically significance higher CD24 expression than #

and † indicates statistically significance higher CD24 expression than ٭.

All at 0.05 significance level

## Discussion

The aim of this investigation was to assess the *CD24* expression and its association with BPH and Gleason grade in prostate adenocarcinoma. In prostate adenocarcinoma patients, *CD24* expression percentage and staining intensity were significantly higher than in BPH patients. The CD24 expression percentage and staining intensity were directly and significantly associated with Gleason grade. The immunoreactive score, which is obtained by multiplying the *CD24* expression percentage with staining intensity, was also significantly related to the Gleason score.

In the year 2022, Borziak and Finkelstein analyzed two sets of single-cell RNA-seq data, focusing on cell types associated with prostate cancer and benign prostatic hyperplasia (BPH). Employing a differential expression analysis encompassing 15,505 epithelial cell profiles and 18,638 genes, the study revealed the overexpression of 791 genes in prostate cancer epithelial cells. The researchers identified six markers exhibiting significant alterations in prostate cancer cells compared to BPH epithelial cells, and among them, CD24 demonstrated a notable increase (2.18X) (17).

In 2019, Chen *et al.* (18) conducted a study to investigate the expression of CD24 in prostate cancer tissue and explore its correlation with the clinicopathological features of patients with prostate cancer. The expression and MFI value (mean fluorescence intensity) of *CD24* were significantly higher in prostate cancer tissues than in prostate paracancerous and benign prostate hyperplasia tissues (18). The findings were in accordance with the present results, which showed a higher expression of *CD24* in prostate cancer patients than in those with BPH. 

In another study, Chen et al performed flow cytometry to detect *CD24* expression on samples from prostate cancer tissue (n=40), paracancerous tissue (n=36), and BPH tissue (n=46). They showed that the expression of *CD24* exhibited a progressive increase in tandem with the advancement of postoperative Gleason score and clinical stage (18). The importance of *CD24* in prostate cancer is not limited to the Gleason score and a study by Cui *et al.* (19). 2021 Indicated that the existence of *CD24* was linked to a risk of metastasis exceeding four times, encompassing both lymph nodes and distant metastases (19). In the current investigation, a direct correlation was observed between the Gleason score and the percentage and intensity of *CD24* staining. These results align with the outcomes reported in earlier studies and indicate the importance of *CD24* for the deterioration of prostate cancer. Intracellular CD24 in prostate cancer cells is found to encourage cell proliferation but inhibit apoptosis, resulting in tumor progression and metastasis, according to a functional analysis (20, 21). This evidence suggests that *CD24* as an oncogene can be an important determinant of prostate cancer aggressiveness.

*CD24* has been identified as a potential prognostic marker in numerous studies across various types of tumors. Specifically concerning prostate cancer, the expression of *CD24* in the cytoplasmic/membrane has been proposed as a means to differentiate between patients with a low or high risk of recurrence (20). Also, in colorectal cancer and non-small cell lung cancer (NSCLCs), the cytoplasmic expression of *CD24* has been identified as an independent prognostic marker for patient survival (20, 21). In bladder cancer, the increased expression of cytoplasmic *CD24* is associated with the more aggressive progression of cancer and the recurrence of tumors (22). Kwon *et al.* (16) demonstrated that the elevated expression of *CD24* in the cytoplasm of breast cancer cells is associated with an adverse prognosis. In the present study, the Immunoreactive score, which is obtained by multiplying the percentage and intensity of *CD24* staining, was also related to Gleason’s score. Therefore, all parameters that were related to *CD24* expression were significantly related to Gleason grade and cancer severity.

## Conclusion

*CD24* expression is a factor that can help distinguish between cases of prostate adenocarcinoma and BPH. The progression of prostate cancer can be indicated by a high level of this marker. Therefore, the evaluation of *CD24* expression can be included as one of the diagnosis options in cases of prostate enlargement.

## References

[1] Zhao J, Xia K, He P, Wei G, Zhou X, Zhang X (2023). Recent advances of nucleic acid-based cancer biomarkers and biosensors. Coord Chem Rev.

[2] Cary KC, Cooperberg MR (2013). Biomarkers in prostate cancer surveillance and screening: past, present, and future. Ther Adv Urol.

[3] Lesko LJ, Atkinson Jr A (2001). Use of biomarkers and surrogate endpoints in drug development and regulatory decision making: criteria, validation, strategies. Ann Rev Pharmacol Toxicol.

[4] Tonry C, Finn S, Armstrong J, Pennington SR (2020). Clinical proteomics for prostate cancer: understanding prostate cancer pathology and protein biomarkers for improved disease management. Clin Proteom.

[5] Doultsinos D, Mills IG (2021). Derivation and application of molecular signatures to prostate cancer: opportunities and challenges. Cancers.

[6] McGrath S, Christidis D, Perera M, Hong SK, Manning T, Vela I (2016). Prostate cancer biomarkers: Are we hitting the mark?. Prostate Int.

[7] Bolton DM, Papa N, Ta AD, Millar J, Davidson AJ, Pedersen J (2015). Predictors of prostate cancer specific mortality after radical prostatectomy: 10 year oncologic outcomes from the Victorian Radical Prostatectomy Registry. BJU Int.

[8] Ortner G, Tzanaki E, Rai BP, Nagele U, Tokas T (2021). Transperineal prostate biopsy: the modern gold standard to prostate cancer diagnosis. Turk J Urol.

[9] Matuszczak M, Schalken JA, Salagierski M (2021). Prostate cancer liquid biopsy biomarkers' clinical utility in diagnosis and prognosis. Cancers.

[10] Schabath H, Runz S, Joumaa S, Altevogt P (2006). CD24 affects CXCR4 function in pre-B lymphocytes and breast carcinoma cells. J Cell Sci.

[11] Lim S-C, Oh S-H (2005). The role of CD24 in various human epithelial neoplasias. Pathol Res Pract.

[12] Zheng Z, Shao N, Weng H, Li W, Zhang J, Zhang L (2015). Correlation between epidermal growth factor receptor and tumor stem cell markers CD44/CD24 and their relationship with prognosis in breast invasive ductal carcinoma. Med Oncol.

[13] Fang X, Zheng P, Tang J, Liu Y (2010). CD24: from A to Z. Cellular Molecul Immunol.

[14] Liu C, Zhang Y, Gao J, Zhang Q, Sun L, Ma Q (2023). A highly potent small-molecule antagonist of exportin-1 selectively eliminates CD44+ CD24-enriched breast cancer stem-like cells. Drug Resist Updat.

[15] Wang Y, Yu H, Yu M, Liu H, Zhang B, Wang Y (2023). CD24 blockade as a novel strategy for cancer treatment. Int Immunopharmacol.

[16] Kwon MJ, Han J, Seo JH, Song K, Jeong HM, Choi J-S (2015). CD24 overexpression is associated with poor prognosis in luminal A and triple-negative breast cancer. PLoS One.

[18] Chen Q, Huang Z, Zhang W, Zheng Z, Sun A Expression of CD24 in prostate cancer tissues and its clinical significance. Chin J Cancer Biother.

[19] Cui X, Wang S, Wang L (2021). CD24 mutant p53 contribute to racial disparities in prostate cancer. Cancer Research.

[20] Weichert W, Denkert C, Burkhardt M, Gansukh T, Bellach J, Altevogt P (2005). Cytoplasmic CD24 expression in colorectal cancer independently correlates with shortened patient survival. Clin Cancer Res.

[21] Karimi-Busheri F, Rasouli-Nia A, Zadorozhny V, Fakhrai H (2013). CD24+/CD38-as new prognostic marker for non-small cell lung cancer. Multidiscip Respir Med.

[22] Liu C, Zheng S, Shen H, Xu K, Chen J, Li H (2013). Clinical significance of CD24 as a predictor of bladder cancer recurrence. Oncol Lett.

